# Modulation of the composite face effect by unintended emotion cues

**DOI:** 10.1098/rsos.160867

**Published:** 2017-04-26

**Authors:** Katie L. H. Gray, Jennifer Murphy, Jade E. Marsh, Richard Cook

**Affiliations:** 1 School of Psychology and Clinical Language Sciences, University of Reading, Reading, UK; 2 Department of Psychology, City, University of London, London, UK; 3 MRC Social, Genetic and Developmental Psychiatry Centre, Institute of Psychiatry, Psychology, and Neuroscience, King's College London, London, UK

**Keywords:** facial emotion, holistic processing, composite-face effect, face perception

## Abstract

When upper and lower regions from different *emotionless* faces are aligned to form a facial composite, observers ‘fuse’ the two halves together, perceptually. The illusory distortion induced by task-irrelevant (‘distractor’) halves hinders participants' judgements about task-relevant (‘target’) halves. This composite-face effect reveals a tendency to integrate feature information from disparate regions of intact upright faces, consistent with theories of holistic face processing. However, observers frequently perceive emotion in ostensibly neutral faces, contrary to the intentions of experimenters. This study sought to determine whether this ‘perceived emotion’ influences the composite-face effect. In our first experiment, we confirmed that the composite effect grows stronger as the strength of distractor emotion increased. Critically, effects of distractor emotion were induced by weak emotion intensities, and were incidental insofar as emotion cues hindered image matching, not emotion labelling *per se*. In Experiment 2, we found a correlation between the presence of perceived emotion in a set of ostensibly neutral distractor regions sourced from commonly used face databases, and the strength of illusory distortion they induced. In Experiment 3, participants completed a sequential matching composite task in which half of the distractor regions were rated high and low for perceived emotion, respectively. Significantly stronger composite effects were induced by the high-emotion distractor halves. These convergent results suggest that perceived emotion increases the strength of the composite-face effect induced by supposedly emotionless faces. These findings have important implications for the study of holistic face processing in typical and atypical populations.

## 1. Introduction

Upright faces are thought to be processed holistically, whereby local facial features are integrated into a unified representation for the purposes of efficient analysis [1–4]. Evidence for this view comes from the composite-face effect [5–7]. When upper and lower regions from different faces are aligned to form a facial composite, the two halves appear to ‘fuse’ together, perceptually. The illusory distortion induced by task-irrelevant (‘distractor’) halves hinders participants' judgements about task-relevant (‘target’) halves (for reviews, see [8,9]). However, when composite arrangements are misaligned spatially, or turned upside-down, the illusion-induced interference is greatly diminished [10,11]. The composite-face effect reveals a tendency to integrate feature information from disparate regions of intact upright faces, consistent with theories of holistic face processing [1–4].

Composite fusion is thought to distort the perception of face structure—a semi-permanent, durable source of facial variation that changes slowly over time [12,13]—leading to biased attributions of facial identity [7], age [14], gender [15] and attractiveness [16]. However, it is well established that manifest expressions—a transient source of facial variation [12,13]—also induce strong composite illusions that interfere with observers' attribution of facial emotion [5,17,18]. For example, observers are error prone and slow when asked to name the emotion of a target half when aligned with a distractor half exhibiting a different emotion, even when the two halves are from the same identity [5].

In recent years, the study of the composite-face effect has been dominated by matching paradigms, whereby observers are asked to judge whether the target regions in two composite arrangements—presented simultaneously or sequentially—are identical or not (e.g. [6,11,19–22]). These procedures are popular because they can be employed with unfamiliar faces (i.e. matching procedures do not necessitate a familiarization phase) and because they allow authors to compare the composite effects seen with faces and other classes of non-face object. Matching paradigms effectively demonstrate the presence of illusory distortion, however, they reveal little about the nature of the distortion induced; the type or direction of illusory bias is ambiguous. The composite-face arrangements employed are constructed from emotionally neutral faces, where the actor depicted has been instructed to convey no emotion. Where observed, composite effects derived from these paradigms are therefore assumed to reflect the binding of facial structure [8,9].

Crucially, however, observers frequently perceive emotion in ostensibly neutral faces, contrary to the intention of the actors themselves *and experimenters* [23]. Capturing valence-free facial expressions is deceptively difficult; when posing for photos, actors seeking to appear ‘neutral’ often appear anxious, bored, threatening or cheerful. In addition, it is not always easy to distinguish a stranger's permanent facial shape from their transient facial expressions [24–26]. For example, it can be difficult to determine whether an unfamiliar actor is sad or simply has a mouth that droops at its corners, whether flared nostrils are a stable facial feature or a display of frustration. Similar effects can also be induced experimentally by feature displacement. For example, simply increasing the vertical distance between the eyes and mouth can augment perceptions of sadness, while decreasing this distance makes the same face appear angry [27].

This study sought to determine how perceived emotion cues influence the composite-face effect. Previous authors have noted that different sets of composite faces produce effect sizes that vary considerably [28,29]. To date, however, little is known about the origin of this inter-stimulus variability. Because image-matching composite paradigms simply require observers to judge whether target halves are identical or not, interference may be induced by the binding of perceived emotion, facial structure or both. Given the strength of the composite effects induced by facial emotion [5,17,18], some of the illusory distortion currently attributed to the binding of facial structure, may in fact be induced by unintended emotion cues [8,9]. Consistent with this possibility, we describe three complementary experiments which suggest that subtle emotions perceived by observers exert a striking influence on the strength of the composite effect.

## 2. Experiment 1

It is well established that emotional distractors impair explicit emotion judgements made about the target region (e.g. labelling or categorization), when arrangements are aligned and upright [5,17,18]. It is unclear, however, whether emotion cues present in the distractor induce ‘incidental’ composite effects; i.e. illusory distortions that affect image matching, in the absence of an explicit emotion judgement. This was the possibility we sought to test in our first experiment. Neutral target regions were presented with task-irrelevant distractor regions, either aligned or misaligned, displaying: (i) no emotion, (ii) weak emotion or (iii) strong emotion. Should distractor regions induce similar levels of illusory interference irrespective of emotion content, it would indicate that the effects obtained using image-matching paradigms reflect the binding of facial structure only. However, modulation of illusory interference by the presence of emotion would imply that emotion cues also induce incidental composite interference.

### 2.1. Material and methods

#### 2.1.1. Participants

Thirty-six naive adults completed the experiment (*M*_age_ = 20.58 years; s.d._age_ = 3.17; eight males). Two participants were replaced having scored 0% correct in one or more of the misaligned conditions. All participants had normal or corrected-to-normal vision. Ethical clearance was granted by the local ethics committee and the study was conducted in line with the ethical guidelines laid down in the 6th (2008) Declaration of Helsinki. All participants gave informed consent.

#### 2.1.2. Stimuli

In all the experiments described, upper face halves were used as target regions, and lower face halves were used as distractors (in line with the prevailing convention [9]). Target regions were taken from 18 neutral faces selected from the Radboud face database [30]. Distractor regions were selected from six different individuals sourced from the same database. Faces were cropped just above the nostrils. Three levels of emotion intensity were produced for each distractor identity, yielding 18 distractors in total. Three of the distractor identities (the happy subset) expressed no emotion, 50% happy and 100% happy. The remaining three distractor identities (the angry subset) expressed no emotion, 50% angry and 100% angry. The 50% intensities were created through image morphing completed using Morpheus Photo Morpher v. 3.11 (Morpheus Software, Indianapolis, IN). Facial composites subtended approximately 6° vertically when viewed at 58 cm. In the misaligned condition, target and distractor halves were offset horizontally by approximately 3°. A thin grey line (approx. 4 pixels) was inserted in between the target and distractor to help participants distinguish the to-be-judged regions. The absence of such delineation may artificially inflate the magnitude of composite-face effects [31].

#### 2.1.3. Procedure

**Figure 1.**
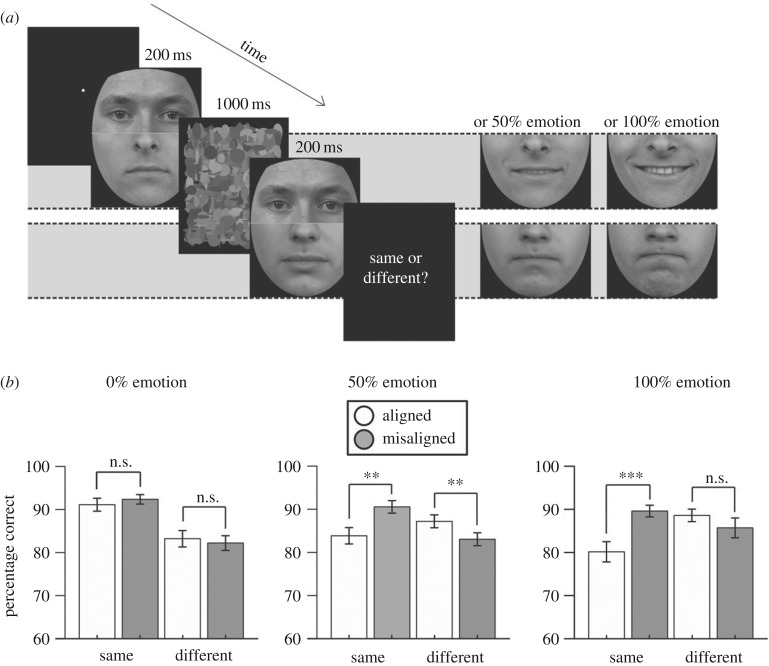
(*a*) Sequentially presented composite faces were presented in which the distractor half either had 0% emotion, 50% emotion or 100% emotion. (*b*) Results from Experiment 1 in the low, moderate and high-emotion conditions. *** denotes *p* < 0.001, ** denotes *p* < 0.01, n.s., non-significant. Error bars denote ±1 s.e.m.

Each trial began with a fixation point, and then presented two composite arrangements sequentially, each for 200 ms (figure 1*a*). During an inter-stimulus-interval of 1000 ms, a mask was presented, constructed from high-contrast greyscale ovals. The target halves could either be identical (50% of trials) or could differ (50% of trials). Participants made simple image-matching judgements about the targets. An original matching design was employed whereby the two distractor halves always differed [6,8]. One distractor was taken from the happy set and one from the angry set (note, this meant that the identity of the distractor always differed). The allocation of happy and angry distractors to the first and second arrangements was counterbalanced. In the no emotion condition, distractor halves had 0% emotion; in the weak emotion condition, distractor halves had 50% emotion; in the strong emotion condition, distractor halves had 100% emotion. Thus, within each trial, the intensity of the expression was held constant, but the actual emotion presented in the two arrangements differed. In total, there were 216 experimental trials: 18 randomly selected target pairings × 2 target types (same, different) × 3 levels of perceived emotion (low, medium, high) × 2 alignments (aligned, misaligned). The different types of trial were randomly interleaved within four blocks of 54 trials. The experiment was programed in Matlab with Psychtoolbox extensions [32,33].

### 2.2. Results and discussion

In the original matching design employed in Experiment 1, distractor halves always differ. The composite illusion is therefore revealed by a disproportionate accuracy cost in the ‘same’ target trials in the presence of aligned distractor regions. Crucially, the interaction between target type (same, different) and alignment (aligned, misaligned) was found to vary as a function of emotion (0%, 50%, 100%) (*F*_2,70_ = 5.50, *p* = 0.006, $\eta_{p}^{2}=0.14$), suggesting that the emotion cues presented in the distractor halves influenced the strength of the composite illusion (figure 1*b*). Evidence of the composite illusion, inferred from simple target type × alignment interactions, was found in both the strong (*F*_1,35_ = 19.11, *p* < 0.001, $\eta_{p}^{2}=0.35$) and weak emotion conditions (*F*1,35 = 16.85, *p* < 0.001, $\eta_{p}^{2}=0.33$), but not in the no emotion condition (*F*1,35 = 1.29, *p* = 0.26, $\eta_{p}^{2}=0.04$). Further analyses revealed that the overall target type × alignment × emotion interaction was driven by differences between the no emotion condition and both the weak (*F*_1,35_ = 7.49, *p* = 0.01, $\eta_{p}^{2}=0.18$) and strong (*F*_1,35_ = 8.48, *p* = 0.006, $\eta_{p}^{2}=0.20$) emotion conditions. The interactions in the weak and strong emotion conditions did not differ significantly (*F*_1,35_ = 0.33, *p* = 0.64, $\eta_{p}^{2}=0.01$).

In the no emotion condition, Bonferroni corrected post hoc contrasts indicated no effect of alignment for either same (*t*_35_ = 0.92, *p* = 0.36) or different trials (*t*_35_ = 0.62, *p* = 0.54). However, accuracy was greater for same trials in both the aligned (*t*_35_ = 3.68, *p* = 0.001) and misaligned (*t*_35_ = 5.05, *p* < 0.001) conditions, suggesting an underlying response bias to respond ‘same’. In the weak emotion condition, we found evidence for a composite effect: observers' accuracy on the same trials was lower when distractors were aligned, than when misaligned (*t*_35_ = 3.34, *p* = 0.002). This effect was reversed for the different trials (*t*_35_ = 3.14, *p* = 0.003). In the strong emotion condition, a classic composite effect was found: once again, observers' accuracy on the same trials was lower when distractors were aligned, than when misaligned (*t*_35_ = 4.90, *p* < 0.001), but this was not the case on different trials (*t*_35_ = 1.32, *p* = 0.20).

These results highlight the striking influence that emotion cues exert on the strength of the composite illusion measured using image-matching paradigms. Previous reports have described how incongruous distractor emotion impairs emotion judgements made about target regions [5,17,18]. However, the present effects of distractor emotion may be thought of as ‘incidental’ insofar as emotion cues hinder image matching, not emotion labelling or categorization *per se*. Importantly, these results confirm that illusion-induced interference seen on image-matching composite procedures may result from the binding of face structure or the binding of facial expression.

Clear and comparable composite illusions were seen when distractor halves depicted strong and intermediate facial emotion. This suggests that the effect is not driven purely by physical dissimilarities in the distractor regions, as the physical differences between strong and intermediate emotion were the same as between intermediate and no emotion conditions. When the distractor halves contained no emotion, however, we found no evidence of a composite effect. We speculate that the lack of a composite effect in this condition may be a product of the procedure employed. Interleaving trials with strong illusory distortion (high emotion) and moderate illusory distortion (intermediate emotion) may have altered participants' decision criteria. While some subtle distortion may be seen in the no emotion condition, it may have been insufficient to elicit ‘different’ responses where participants have the reasonable expectation that ‘same’ and ‘different’ responses should be made with roughly equal frequency within a block.

## 3. Experiment 2

The results of Experiment 1 confirm that emotion cues present in distractor regions may induce incidental composite interference, impairing image-matching judgements made about target face regions. Moreover, it appears that relatively weak emotion cues present in the distractor regions are sufficient to induce target distortions. In our second experiment, we sought evidence that perceived emotion in ostensibly ‘neutral’ faces might modulate composite interference in a similar way. If perceived emotion modulates composite binding, distractor halves rich in perceived emotion should exert more illusory distortion on target halves. In Experiment 2, we therefore examined the relative ability of 50 distractor halves—all supposedly ‘neutral’—to distort observers' perception of four target halves, to determine whether this variability is associated with the presence of perceived emotion. Traditional composite-face procedures collapse across multiple targets and distractors to derive a single estimate of observers' susceptibility to the illusion. To estimate the composite interference induced by individual distractors, we therefore employed a novel subjective-report paradigm.

### 3.1. Material and methods

#### 3.1.1. Participants

The emotion rating task was completed by 30 naive adults (*M*_age_ = 30.8 years; s.d._age_ = 8.0; nine males). A separate group of 46 nave adults (*M*_age_ = 46.3 years; s.d._age_ = 9.1; 16 males) participated in the composite distortion task. All participants had normal or corrected-to-normal vision. Ethical clearance was granted by the local ethics committee and the study was conducted in line with the ethical guidelines laid down in the 6th (2008) Declaration of Helsinki. All participants gave informed consent.

#### 3.1.2. Stimuli

The 50 distractor halves and four target halves were cropped from 54 male faces sourced from the Karolinska Directed Emotional Faces [34] and the Radboud Faces Database [30]. Importantly, each actor depicted was posing a neutral expression (i.e. trying not to convey facial emotion). External facial features were occluded using an oval frame. Faces were cropped just above the nostrils. The distractor and target halves were presented in greyscale against a mid-grey background. Once again, a thin grey line (approx. 4 pixels) was inserted in between the target and distractor to help participants distinguish the to-be-judged region. Participants in the rating phase were required to rate each of the 50 distractors for the presence of five emotions (happiness, anger, fear, sadness and disgust)^1^ on a 1–100 scale. Each rating trial presented a single distractor in isolation.

#### 3.1.3. Procedure

Two identical target halves were presented on the left and right side of the display, separated by approximately 7° of visual angle when viewed at 58 cm. On each trial, the left-hand target was aligned with one of the 50 distractors to create a facial composite subtending approximately 6° vertically. The right-hand target was always presented in isolation. Having been told that the targets were physically identical, participants were required to report the strength of the distortion induced by the distractor using a slider (from no distortion to substantial distortion, on 1–50 scale). No time limit was imposed. Each distractor region was paired with four different eye regions, resulting in 200 subjective-report trials, completed in a randomized order. To help participants familiarize themselves with the nature and strength of the illusion, they viewed all 200 displays for 3 s each before starting the rating procedure. We hoped pre-exposure would improve participants' ability to describe the relative strength of the distortion on a given trial. The experiment was programed in Matlab with Psychtoolbox extensions [32,33].

### 3.2. Results and discussion

**Figure 2.**
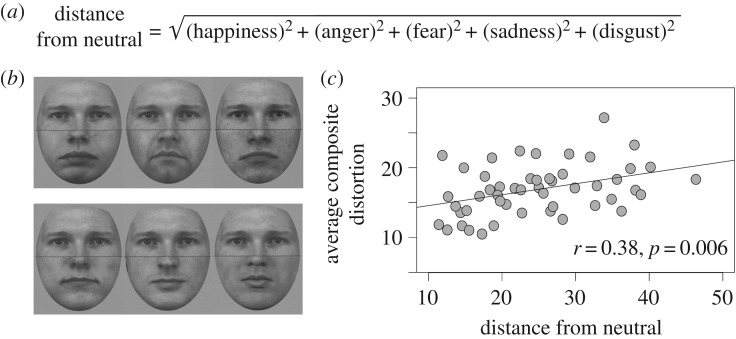
(*a*) To produce a single estimate of the perceived emotion present in each distractor, we computed its Euclidean distance in emotion space from the point of absolute neutrality. (*b*) Examples of facial composites used Experiment 2 constructed with distractors rated high (top) and low (bottom) in perceived emotion. Observers were required to rate the extent to which the lower face half distorted their percept of the upper face half. (*c*) The correlation between the average magnitude of composite distortion and the average distance of each distractor half from true neutral.

The subjective reports of illusory distortion induced by the distractors, provided by each participant, were first averaged across the four target halves (to derive the average distortion reported by a given participant, for each distractor), then averaged across participants (to compute the average distortion reported by the sample, for each distractor). To produce a single measure of the perceived emotion present in each distractor, we calculated its Euclidean distance in emotion space^2^ from the point of absolute neutrality (figure 2*a*). Smaller scores indicate that distractors were rated closer to neutral and therefore contained less perceived emotion. Despite being cropped from ostensibly emotion-neutral faces, there was considerable variability in the mean distances computed (figure 2*b*).

Simple correlation analysis (figure 2*c*) revealed a significant positive relationship (*r* = 0.38, *p* = 0.006) between the degree of perceived emotion (*M* = 24.73, s.d. = 8.77) and the composite distortion induced by the distractors (*M* = 16.86, s.d. = 3.60). Consistent with the view that perceived emotion in supposedly neutral faces induces incidental composite effects, distractors rated as more emotional induced stronger illusory distortion. To our knowledge, this is the first attempt to understand stimulus-specific variation in composite interference.

The first two experiments employed complementary approaches to study the role of subtle emotion cues on the composite effect; artificially introducing an emotion signal using image morphing (Experiment 1), and using the natural variation present in the population of ‘neutral faces’ available in commonly used face databases (Experiment 2). Nevertheless, the results are convergent; relatively subtle emotion cues, either intended or unintended, can exert a striking influence on the strength of target distortions induced by the composite illusion.

## 4. Experiment 3

The results from Experiment 2 suggest a relationship between the emotion ratings awarded to ostensibly neutral distractors and the degree of composite distortion induced. However, describing one's subjective experience of an unfamiliar illusion is challenging [37]. In Experiment 3, we therefore sought to determine whether variation in perceived emotion present in ostensibly neutral faces also modulates performance on a sequential matching composite procedure. In Experiment 1, we employed the original matching design, whereby the distractor regions always differ [8]. However, in Experiment 3, we employed a congruency procedure that also included trials where distractors were the same. Some authors have speculated that this design measures composite-face effects in a way that attenuates the influence of response bias [38] (for a different view see [8]). For the sake of clarity, we provide supplementary analyses of those trials where the distractors differ (the original design). We note, however, that these results suggest a similar conclusion to those obtained with the full congruency design. An inverted control condition was also employed to confirm that the effects of alignment are orientation sensitive [10].

### 4.1. Material and methods

#### 4.1.1. Participants

Twenty naive adults (*M*_age_ = 27.2; s.d._age_ = 4.7; five males) with normal or corrected-to-normal vision participated in Experiment 3. Ethical clearance was granted by the local ethics committee and the study was conducted in line with the ethical guidelines laid down in the 6th (2008) Declaration of Helsinki. All participants gave informed consent.

#### 4.1.2. Stimuli

Eighteen distractor halves were selected from the 50 used in Experiment 2. The nine judged closest to true neutral were selected for use in the low perceived emotion condition, and the nine judged furthest from true neutral were selected for use in the high perceived emotion condition. Once more, we note that all 18 were cropped from supposedly ‘neutral’ faces. Eighteen target halves were sourced from the Karolinska [34] and Radboud databases [30], including the four used in Experiment 2. Facial composites subtended approximately 6° vertically when viewed at 58 cm. A thin grey line (approx. 4 pixels) was inserted in between the target and distractor to guide participants' judgements.

#### 4.1.3. Procedure

**Figure 3.**
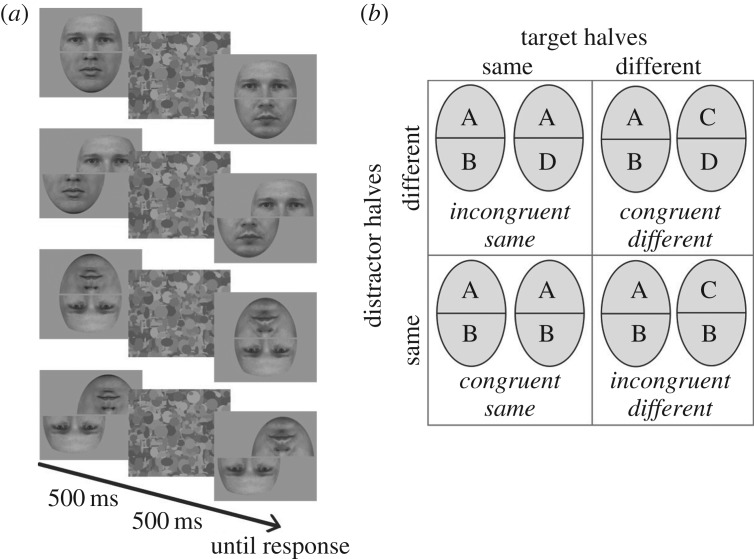
(*a*) Illustration of each condition; upright aligned, upright misaligned, inverted aligned and inverted misaligned. Each trial began with a central fixation cross. The first target face was then presented for 500 ms, followed by a mask for 500 ms. The second face was visible until a response was registered. Observers responded ‘same’ or ‘different’ using the keyboard. Observers were instructed to make their judgement on the upper face half (i.e. the eye region) irrespective of composite orientation. (*b*) Trial types for the complete composite design used in Experiment 3.

On each trial, observers were asked to indicate whether the target halves of two sequentially presented composites were the same or different, in the presence of distractor halves that were either identical or different, and either high or low in perceived emotion. Two control manipulations were employed; an inverted condition, where both composites were presented upside-down, and a misaligned condition, where target and distractor halves were offset horizontally by approximately 3° (figure 3*a*). In total, there were 576 experimental trials: 18 target combinations × 2 target types (same, different) × 2 distractor types (same, different) × 2 levels of perceived emotion (high, low) × 2 orientations (upright, inverted) × 2 alignments (aligned, misaligned). All trial types were randomly interleaved. The experiment lasted 35 min and was separated into 10 blocks. The experiment was programed in Matlab with Psychtoolbox extensions [32,33].

Presenting every possible target-distractor pairing in each of the different conditions would have necessitated a prohibitive number of experimental trials. While everyone judged the same 18 target combinations on the same trials (i.e. all 18 target halves), each participant judged a different set of 18 target combinations on the different trials. Distractor halves were assigned pseudo-randomly. Where different distractors were employed on a trial, they were chosen from the same emotion condition (half high perceived emotion; half low perceived emotion). For 50% of the same trials, the targets were paired with the same distractor (congruent-same trials); for the remaining same trials, targets were paired with different distractors (incongruent-same trials). For 50% of the different trials, targets were paired with the same distractor (incongruent-different trials); for the remaining different trials, targets were paired with different distractors (congruent-different trials).

### 4.2. Results and discussion

Target halves (*T*_same_, *T*_different_) and distractor halves (*D*_same_, *D*_different_) were combined in a complete factorial design, yielding four possible trial types (figure 3*b*): congruent-same (*T*_same_, *D*_same_), incongruent-same (*T*_same_, *D*_different_), congruent different (*T*_different_, *D*_different_), and incongruent-different (*T*_different_, *D*_same_). On congruent trials, composite effects are thought to aid observers' performance (identical distractors facilitate ‘same’ decisions about identical targets; different distractors facilitate ‘different’ decisions about non-identical targets). On incongruent trials, composite effects are thought to impair observers' performance (identical distractors hinder ‘different’ decisions about non-identical targets; different distractors hinder ‘same’ decisions about identical targets). In this congruency design, composite effects are therefore indexed by a disproportionate effect of congruency (congruent, incongruent) when composites are upright and aligned, relative to inverted or misaligned conditions (e.g. [21,39]). For each cell in the design (emotion × orientation × alignment), we therefore estimated observers' discrimination sensitivity on congruent and incongruent trials through the calculation of *d'* statistics [40].

**Figure 4.**
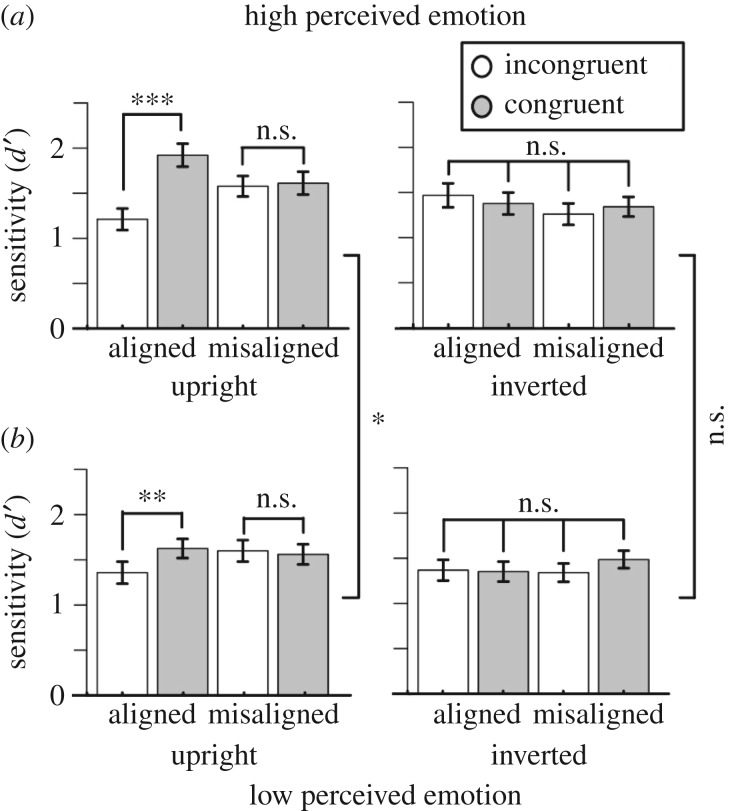
Results from Experiment 3 in the (*a*) high, and (*b*) low perceived emotion conditions. *** denotes *p* < 0.001, ** denotes *p* < 0.01, * denotes *p* < 0.05, n.s., non-significant. Error bars denote ± 1 s.e.m.

Significant composite effects, indicated by characteristic congruency × orientation × alignment interactions, were seen in both the high (*F*_1,19_ = 22.23, *p* < 0.001, $\eta_{p}^{2}=0.54$) and low (*F*_1,19_ = 4.53, *p* = 0.047, $\eta_{p}^{2}=0.19$) perceived emotion conditions (table 1 and figure 4). Critically, however, composite effects were larger when distractors contained high levels of perceived emotion (*F*_1,19_ = 4.56, *p* = 0.046, $\eta_{p}^{2}=0.41$). This interaction with emotion was driven by sensitivity differences in the upright conditions, indicated by a significant emotion × congruency × alignment interaction (*F*_1,19_ = 6.25, *p* = 0.02, $\eta_{p}^{2}=0.25$). When composites were upright and aligned there was also an emotion × congruency interaction (*F*_1,19_ = 9.30, *p* = 0.007, $\eta_{p}^{2}=0.33$). Importantly, none of the interactions with emotion reached significance when composites were inverted or misaligned (all *F*'s < 2.6; all *p*'s > 1.2). When the composites were presented upright and aligned, effects of congruency were observed in both the high (*t*_19_ = 6.0, *p* < 0.001) and low (*t*_19_ = 2.98, *p* = 0.008) perceived emotion conditions. Observers' sensitivity differed significantly for the high and low emotion distractors on the congruent trials (*t*_19_ = 3.08, *p* = 0.006), but not on the incongruent trials, (*t*_19_ = 1.29, *p* = 0.21).

**Table 1.** Results of the ANOVAs performed on the high and low perceived emotion conditions in Experiment 3. (Values in bold indicate significant values.)

	high perceived emotion			low perceived emotion		
	*F*	*p*	$\eta_{p}^{2}$	*F*	*p*	$\eta_{p}^{2}$
congruency	2.51	0.130	0.12	4.29	0.052	0.18
orientation	**14**.**28**	**0**.**001**	**0**.**43**	**10**.**69**	**0**.**004**	**0**.**36**
alignment	0.09	0.767	0.01	3.93	0.062	0.17
congruency × orientation	**12**.**86**	**0**.**002**	**0**.**40**	1.23	0.282	0.06
congruency × alignment	**5**.**80**	**0**.**026**	**0**.**23**	0.15	0.710	0.01
orientation × alignment	0.078	0.784	0.01	0.09	0.770	0.01
congruency × orientation × alignment	**22**.**23**	<**0**.**001**	**0**.**54**	**4**.**53**	**0**.**047**	**0**.**19**
upright						
congruency	**9**.**45**	**0**.**006**	**0**.**33**	**5**.**15**	**0**.**035**	**0**.**21**
alignment	0.150	0.703	0.01	2.51	0.130	0.12
congruency × alignment	**16**.**91**	**0**.**001**	**0**.**47**	2.12	0.162	0.10
inverted						
congruency	2.72	0.120	0.13	0.63	0.438	0.03
alignment	0.004	0.950	0.00	1.53	0.230	0.07
congruency × alignment	2.07	0.170	0.10	3.44	0.079	0.15

## 5. General discussion

The findings from these complementary experiments indicate that subtle facial emotion cues exert a striking influence on the strength of the composite-face effect. In our first experiment, we found that composite interference grew stronger as the strength of the emotion signal present in the distractor increased. Critically, effects of distractor emotion were induced by relatively weak cues (only 50% of the full emotion intensity), and were incidental insofar as emotion cues hindered image matching, not emotion labelling or categorization *per se*. Next, we examined whether perceived emotion cues present in ostensibly neutral faces, are strong enough to modulate composite interference in a similar way. We found a correlation between the strength of perceived emotion cues (rated by one set of participants) and the strength of illusory distortion induced (assessed by different participants) in a set of 50 ‘neutral’ distractors taken from commonly used face databases. In Experiment 3, we compared the composite effects induced by ostensibly neutral distractors rated high and low for perceived emotion, measured using a sequential matching task. We found significantly larger composite effects were induced by the emotion-rich distractors; strikingly, the characteristic interaction effect was more than twice as strong in the high perceived emotion condition.

Different learning traditions have converged on the principle that the degree of covariation between stimulus elements determines whether they will be grouped together [41,42]. Crucially, facial emotions are known to comprise highly correlated feature changes [43]. Exposure to this covariation may therefore provide a strong basis for inter-feature perceptual prediction [44,45], and underlie the compelling composite distortion induced by facial emotion [5,17,18]. The identity [7], age [14] and gender [15] composite effects may have a similar origin; for example, in our day-to-day environment, the presence of a male mouth reliably predicts the presence of male eyes. We speculate that the strength of illusory distortion induced by different composite arrangements may be determined by the strength of these cross-feature contingencies.^3^ Subtle expression cues may exert a strong influence on the composite-face effect because of the striking statistical regularities seen in facial expressions [43]. We note that composite effects have recently been reported with expressive body postures [46], but not for neutral body shapes [47]. The highly coordinated nature of whole body actions may also underlie the composite effects seen in this domain.

The present results suggest that composite effects measured in sequential image-matching paradigms probably reflect illusory interference induced by both expression and structure cues. We are not in a position to determine whether these sources of distortion interact or combine additively. Insofar as facial structure and facial expression are largely independent sources of facial variation [48,49], perceptual predictions derived from structure and expression cues may also be relatively independent [5]. Nevertheless, illusory distortion induced by expression cues may hinder the matching of targets based on facial *identity*. Observers experience well documented difficulties encoding the facial structure of unfamiliar faces [50–52]. For example, when asked to sort photographs of two unfamiliar individuals according to the identity of those depicted, observers perform poorly, frequently attributing the photographs to eight or more different individuals [53]. Deriving an expression-invariant description of unfamiliar faces poses a particular challenge; when viewing a single image, it is often impossible to determine whether a stranger is scowling or has narrow eyes. In light of the difficulties partitioning facial variance according to structure and expression, expression distortions may affect identity matching for unfamiliar faces.

Previous research has revealed that perceived emotion can exert a strong influence on the judgements we make about the character traits of others. For example, the detection of anger and happiness may be responsible for trait judgements of dominance and trustworthiness inferred spontaneously from supposedly neutral faces [26,54]. Consistent with this view, observers who have difficulties interpreting facial emotion, make unusual trait judgements about neutral faces [55]. The current findings further illustrate the unexpected effects that unintended emotion cues may exert on the perception of ‘emotionless’ faces. Such cues may not only influence the judgement of character traits but may modulate the extent to which faces are processed holistically. Interestingly, the present findings suggest the possibility that highly trustworthy and highly dominant faces may tend to produce large composite effects, insofar as both may be rich in perceived emotion cues (see also [56]).

We have argued that subtle emotion cues present in distractor regions exert a striking influence on the strength of composite-face effects, possibly because of the strength of the inter-feature contingencies present in manifest facial expressions. However, some readers might query whether emotion cues modulate composite effects via another route. If the presence of emotion cues made the distractor regions more salient, they may have impaired matching through generic distraction, rather than distortion induced by the composite-face illusion. Two of our findings speak against this alternative account. First, generic distraction effects should be relatively insensitive to the alignment manipulation. Crucially, however, we only saw effects of emotion when distractor regions were aligned; the presence of emotion had little effect when distractors were misaligned. Second, effects of emotion were seen when participants were asked to rate the strength of the illusory distortion without any time pressure (Experiment 2). Distraction effects might conceivably impair sequential matching ability where arrangements are presented very briefly. In Experiment 2, however, participants could take as long as they wished to compare the target aligned with the distractor, and the target presented in isolation.

A further possibility that warrants discussion is the suggestion that the increased strength of the composite illusion was not attributable to facial emotion *per se*. Instead, some distractor regions with unusual or distinctive facial structure were perhaps more likely to be perceived as emotional; for example, ambiguous face shapes may be more receptive to a high-emotion perceptual interpretation. Thus, apparent modulation by facial emotion may have been driven by underlying facial structure variation. Again, however, features of our data speak against this view. First, in Experiment 1 we found that increasing the strength of the emotion signal present on the same facial identities can increase the strength of the composite illusion. In this situation, there is little possibility that perceived emotion is confounded with facial structure. This finding confirms that effects of emotion can be seen independently of facial structure. Second, it is evident from Experiment 2 that perceived emotion cues are present in a great many ‘neutral’ distractor regions sourced from popular face databases. It seems unlikely that all of these faces are unusual or distinctive. Rather, it appears that posing expressions which are truly emotion neutral may be a formidable challenge for actors of all face shapes.

**Figure 5.**
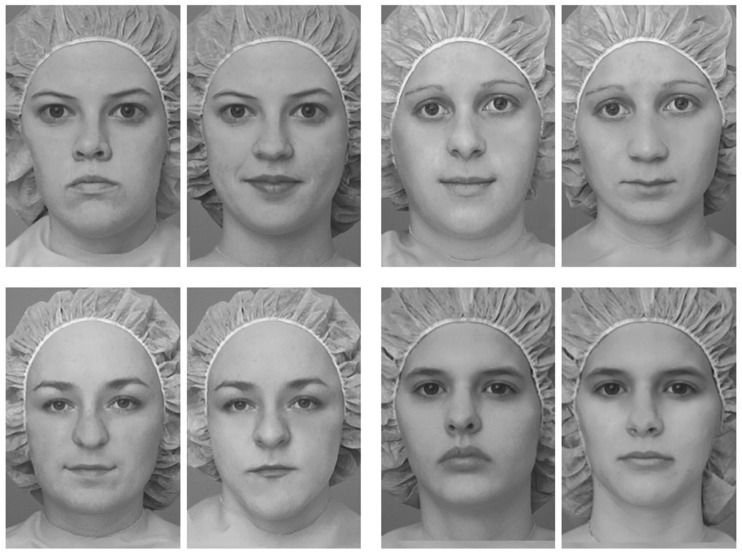
Examples of facial composites taken from a popular stimulus set developed by Le Grand and co-workers [20]. This set has been widely used to investigate holistic processing in typical and atypical populations [17,22,63–66]. While composites are constructed with ostensibly neutral faces, subtle emotion cues are present in many of the arrangements. These unintended emotion cues, together with the absence of a gap between the target and distractor regions [31], may contribute to the large effect sizes seen with this set.

The present results have important implications for researchers using the composite-face paradigm to investigate holistic processing in typical and atypical populations. Previous studies comparing individuals' susceptibility to the composite illusion and other markers of holistic processing, notably the part-whole effect [57], have yielded inconsistent findings [58–60]. The relationship between observers' susceptibility to the composite-face effect and their face recognition ability also remains uncertain[22,58–62]. These mixed results have cast doubt on the functional significance of holistic face processing as measured by the composite paradigm [9]. Crucially, however, many widely used stimulus sets contain composites rich in facial emotion (figure 5). While these sets may yield strong replicable composite effects, individual differences may be less likely to correlate with susceptibility to the part-whole effect and measures of face recognition ability. Instead, the present results raise the possibility that individual differences in illusion susceptibility may sometimes correlate with measures of *expression* recognition.

The present results also have implications for the study of holistic face processing in atypical populations. For example, some authors have found that observers with autism spectrum disorder (ASD) exhibit broadly typical composite-face effects [63], whereas other findings indicate abnormal processing of facial composites [67]. Importantly, however, there is considerable heterogeneity within the ASD population in terms of expression recognition [68,69]. This variability may help explain the inconsistent performance of ASD samples on composite-face tasks. Similarly, cases of developmental prosopagnosia (DP) have been described who exhibit typical composite effects despite severe face recognition difficulties [64,70]. However: (i) it is known that many DPs exhibit good expression recognition [71,72], and (ii) the composite stimuli used in these studies include salient emotion cues. It is unclear, therefore, whether these individuals exhibit intact holistic face processing *per se*, or intact holistic processing of facial emotion.

Finally, several authors have sought to investigate the origin and specificity of the composite effect by comparing the strength of illusory interference induced by faces and other types of object [73]. However, the strength of the *face* composite effect will probably depend on the degree of perceived emotion present in the arrangement. When contrasting the size of composite effects induced by the binding of face shape with those seen for rigid non-face objects-of-expertise, such as ‘Greebles’ [74], authors should seek to exclude perceived emotion cues from their face arrangements; i.e. to ensure binding is based solely on the covariation of structure cues in the stimulus classes compared. We speculate that animating to-be-learned items with coordinated patterns of global change—mirroring the correlated dynamics of whole body actions and facial expressions—may increase the strength of composite interference seen with non-face objects-of-expertise.

The results from the three experiments described indicate that perceived emotion cues modulate the strength of the composite-face effect when stimulus arrangements are constructed from supposedly ‘neutral’ faces. These results have important implications for research addressing holistic processing in typical and atypical populations. Understanding the contribution of perceived emotion to inter-stimulus variability may help reveal the relationship between composite interference, other markers of holistic face processing, and face recognition ability.

## Supplementary Material

Data

## Supplementary Material

Supplementary analyses of Experiment 3

## Footnotes

1 Surprise was not included as an option in the rating task, as the status of surprise as a basic emotion has been questioned (e.g. [35]). There is also some evidence that the perceptual representation of surprise behaves differently to that of other facial emotions; e.g. surprise expressions may not be perceived categorically [36].

2 The use of Euclidean distances assumes orthogonal dimensionality. When judging whole-face expressions, ratings of anger and disgust are known to correlate. Some sign of this association was also seen in the half-face ratings collected in Experiment 2; however, the correlation was not strong (*r* = 0.32).

3 Upper face halves are commonly used as distractor regions in composite paradigms insofar as mouth-to-eye interference is typically far stronger than eye-to-mouth interference. Interestingly, this asymmetry potentially accords with a feature covariation account. Predictions made about the eye region based on the state of the mouth region may be more reliable than the predictions made about the mouth region based on the state of the eye region. By way of comparison, when English readers encounter the letter ‘Q’ in a sentence, there is a strong likelihood that the next letter will be ‘U’. Conversely, when the letter ‘U’ is encountered, it is less likely that the preceding letter will be ‘Q’.

## Data accessibility

The dataset supporting this article are available as the electronic supplementary material.
