# Supplementary material for: Modulation of the composite face effect by unintended emotion cues
Source: R Soc Open Sci. 2017 Apr 26;4(4):160867. doi: 10.1098/rsos.160867 (PMC5414244; doi:10.1098/rsos.160867)
Supplement: Supplementary analyses of Experiment 3 [file rsos160867supp2.docx]

*Supplementary analyses for:*

Modulation of the composite face effect by unintended emotion cues

Katie L.H. Gray, Jennifer Murphy, Jade E. Marsh, Richard Cook

Below we describe additional analyses conducted on Experiment 3 to determine whether our key finding – stronger composite effects in the high perceived emotion condition – was also evident when we considered only those cells included in the original design (i.e., incongruent-same and congruent different trials). Here, as in Experiment 1, a composite effect is indexed by a disproportionate accuracy cost in the ‘same’ target trials in the presence of an aligned distractor.

Crucially, our analysis indicated that the Target Type (same, different) × Alignment (aligned, misaligned) interaction varied as a function of perceived emotion (High, Low) [*F*(1,20) = 6.5, *p* = .019, η*_p_*^2^ = .25] (see Figure S1). There was clear evidence of a simple Target Type × Alignment interaction in the high perceived emotion condition [*F*(1,20) = 30.24, *p* < .001, η*_p_*^2^ = .60], but only marginal evidence for a Target Type × Alignment interaction in the low perceived emotion condition [*F*(1,20) = 3.77, *p* = .07, η*_p_*^2^ = .16].

In the high perceived emotion condition, Bonferroni corrected post-hoc contrasts revealed lower accuracy for aligned ‘same’ trials than for misaligned ‘same’ trials [*t*(20) = 2.86, *p* = .01]. This effect was reversed for different trials [*t*(20) = 3.93, *p* < .001]. In the low perceived emotion condition, there was a trend towards lower accuracy for aligned ‘same’ trials than misaligned ‘same’ trials [*t*(20) = 1.80, *p* = .09], but there was no difference in the accuracy of aligned and misaligned different trials [*t*(20) = 1.16, *p* = .26].

These additional results confirm that perceived emotion modulates the strength of the composite face effect measured using either the congruency or the original variants of the matching task.


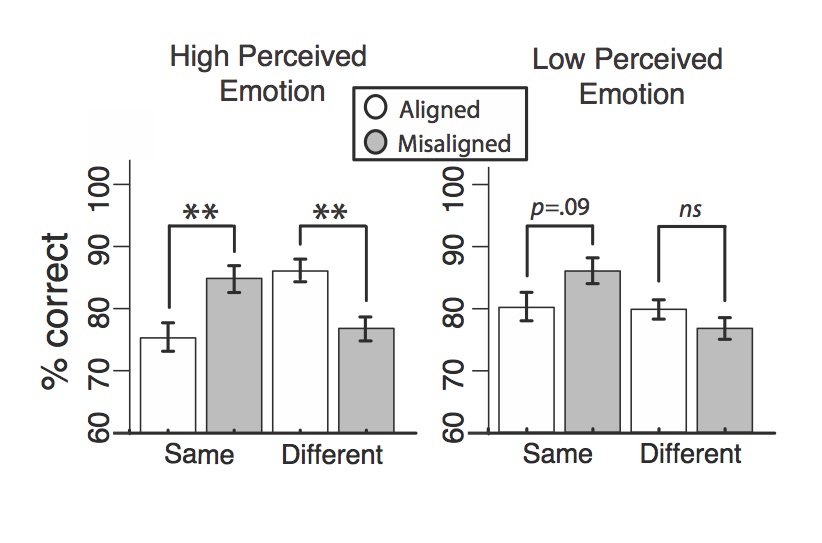


Figure S1. Results from supplementary analyses of Experiment 3 in the high and low perceived emotion conditions. *** denotes *p* < .001, ** denotes *p* < .01, * denotes *p* < .05. Error bars denote ±1SEM.
